# Annotations

**Published:** 1901-03-16

**Authors:** 


### ANNOTATIONS.

### Infection by Means of Clothing.

Few ideas are more strongly fixed in the public mind than the notion that infection is carried by clothes. Many wonderful tales are told about bits of flannel, old petticoats, and other articles of clothing, which, after being used in the nursing of scarlet fever, have been put away for untold years, only to set up fresh outbreaks of disease when at last dug out by careful housewives from some long undisturbed hiding places, and the evidence, although often somewhat roundabout, has sometimes seemed sufficient to support the theory. The daily life, however, of every doctor appears to give the lie to any such idea. We see the doctor on his round popping into one house after another, and it is hardly to be wondered at that the man in the street—always a critical person, and sometimes lucky enough to hit the right nail on the head—should now and again declare that for a doctor to jump into his brougham and wrap himself up in his furs after visiting an infectious case is hardly consistent with the little homilies that he has just been reading to the patient's friends about their lack of consideration for others in going out shopping and paying calls while they have fever in the house! For our comfort, however, Dr. Doty, health officer of the Port of New York, well known for his investigations in regard to infectious diseases and for his admirable administrative work, has recently read a paper before the American Public Health Association in which he held that infectious diseases are rarely communicated by means of clothing. This he says is not a mere theoretical conclusion, but is the result of observations made during a continued experience of 20 years with infectious disease as health officer; and he adds: "As a matter of fact these views are apparently endorsed by the medical profession both in private practice and in matters relating to public health, inasmuch as physicians daily visit infectious diseases and go from them directly to other patients without disinfection or change of clothing. Moreover, health departments throughout the country permit their inspectors and diagnosticians to visit infectious disease in the same manner. . . . In families where scarlet fever exists the adult members, who are actively employed outside, are allowed to continue their business without interruption. Of course they are usually admonished not to enter the apartment of the sick when at home, but in a large percentage of cases the patient roams about the house or apartment at will. Therefore, if the clothing worn by well persons were a medium of infection to the extent which is commonly believed, we would certainly and surely have indisputable evidence of it, which we do not." Dr. Doty however admits that infection "may in some cases be transmitted through the medium of the clothing of well persons," but he holds quite strongly that this does not commonly occur, and that in making rules and regulations for the protection of the public health we must not give undue consideration to such a possibility. We think that on the whole Dr. Doty is right. We are quite clear about the possibility of infection being sometimes carried by fomites; in fact, to deny this in cases where the fomites are soiled by the proper stuff and preserved in the proper manner to ensure transmission would be to deny the whole theory of infection. On the other hand it is obvious enough that infection by the clothing of "well people" only rarely occurs, and we take it that in this matter the element of time and the amount of exposure have much to do with the result. We are constantly being asked by nurses, "Why all this fuss when the doctor goes in and out without taking any precautions?" and if we were to admit the theory of mediate contagion in its extreme degree it must be confessed that no answer would be forthcoming. But we must consider the shortness of the doctor's visit and the comparatively small opportunity of direct infection of his clothes, compared with the prolonged exposure and intimate contact with the patient which occurs in the case of the nurse. Still it must be confessed that the possibility of infection being carried by the medical attendant has always been somewhat of a nightmare to us, and although it is a relief to find that Dr. Doty with his undoubtedly extensive experience is able to speak as strongly as he does, from the practical point of view, about the improbability of infection being often carried from case to case in the clothing of "well persons," we cannot but feel a certain sympathy with the "walking doctor," or the one who on horseback, or on cycle, or even in an ordinary "doctor's gig," does at least get some aëration between his cases; and, *per contra*, a little doubt about the comfortable person who, in furs and brougham, carries with him little whiffs of sick-room atmosphere from case to case.

### Lunacy Law.

For several years past we have been accustomed to see a Lunacy Bill make its appearance before one or other House of Parliament, only to be withdrawn or to dissolve away after short discussion. Now, however, that we find a Bill for the better administration of the law respecting lunatics mentioned in the King's Speech as having been prepared to be laid before Parliament, we may look forward with more confidence to the passing of some such measure. A very serious defect in the Bill of last year was the omission of all provision for ensuring that medical superintendents should obtain proper pensions on retirement after their full term of service, and it is to be hoped that this will be rectified in the coming Bill of this year. To the public and to those medical men outside the speciality the point in the Lunacy Law which appears most urgently to demand reformation, is that which, by drawing a sharp line between sanity and insanity, leaves those unfortunates who cannot be classed under either category practically deprived of hearth and home. In the Lunacy Bill of the year before last, the Lord Chancellor introduced a clause, which appeared again in the Bill of last year, giving facilities for placing incipient and non-confirmed cases in private homes for six months without the formalities required under ordinary circumstances. There can be no doubt that some such provision is necessary for the smooth working of the law. It is constantly the case that a very considerable number of people who are insane, but are as yet not certifiable, are being illegally received for payment, sometimes as private patients, sometimes as visitors to hydropathic institutions, sometimes merely as boarders by lodging-house keepers. According to the law as it stands all this is illegal. But some means ought to be devised by which these incipient and non-confirmed cases could legally be taken care of pending the time when they either recover or become certifiable. This the Bill of last year proposed to do, and it is to be hoped that the Bill which is to be brought forward this session will contain some provision with the same object.

### Temperance Reform in Kansas.

Temperance reform is being pushed vigorously in the State of Kansas. "Vigorous" is the word that best describes the methods of the reformers. The leader among them is a lady, Mrs. Carrie Nation, who has certainly achieved notoriety, if not greatness, by her efforts. Her methods of attacking the evil of drunkenness are simple and direct. She goes straight and attacks the publichouse, or saloon, as the Americans call it. She smashes the windows and, assisted by sympathetic lady friends, does all possible damage to the place. Recently Mrs. Nation addressed a circular letter to the publicans of the town in which she lives, which began, "My Dear Hell-bound Sinners." The letter thus startlingly begun contained, however, nothing more formidable than a request that the "Hell-bound Sinners" would appoint a time and place where she might meet them and discuss the drink question. She mentioned, however, that if they could not come to a satisfactory arrangement with her, forcible measures would be taken to make them shut up their premises. To us it seems that such conduct, even when actuated by the highest motives, would soon land the window-smasher in gaol; but it seems that it is still undecided whether the law of Kansas protects saloon property. The owner of one building, however, where the "Nationites" as they are called, had wrought havoc has laid a suit for damages against the lady who injured his property. She is a Mrs. Sheriff, and is well off, so she has engaged a good lawyer to defend her. It remains to be seen what view a Kansas judge will take of the case. Similar attacks are being made in other places. In Sioux City a band of women went down and broke everything they could lay hands on in one saloon. One lady tore down a brewer's sign, while another painted out the word "saloon" with white paint. Having finished with one place they proceeded to another, but here they found the proprietor at the door armed with a revolver. He informed them that if they entered the place it would be over his body, so that attempt was abandoned. In another case it was the saloon-keeper's wife who armed herself, and threatened to make trouble among her temperance sisters if they dared to invade her husband's premises.

### Smallpox in Scotland.

The prospect which is before the City of Glasgow of having its exhibition ruined by the continued prevalence of smallpox is not a pleasant one, and must be all the more galling to the decent citizens from the fact that the majority of them have already taken the precaution of submitting to re-vaccination, and that it is only the obstinacy of the stupid minority which stands in the way of the exhibition, to the organisation of which much money and great trouble have been given, becoming a great success. In view of the fact that far more than half the adult population of Glasgow have already been re-vaccinated, we can hardly doubt that if they had local option they would soon make re-vaccination compulsory on everyone. But that cannot be, and Glasgow, we are afraid, will have to be content to pose as a standing example of the inefficiency of sanitary measures which cannot be made obligatory on all alike. Every effort is no doubt being made; but voluntary vaccination always leaves a residuum of susceptible persons, and we can hardly expect that in the short time now remaining before the time of the opening of the exhibition in May an outbreak so extensive as that which has for many months been in progress in Glasgow will be brought under control. Unfortunately Glasgow is in intimate communication by way of its large working population with most parts of Scotland, and it is much to be feared that so severe a manifestation of smallpox will not long remain confined to that city. Cases have already occurred in Edinburgh, and it is by no means likely that we have yet got to the end of the story.

### The Birmingham Consultative Institute.

The resignation by Dr. Irvine of his connection with the Birmingham Consultative Institute has landed the managers of that institution in a considerable difficulty; for if we may judge from the tone of the local press the people of Birmingham are beginning at last to understand what has been pointed out to them again and again, that if they want expert advice of a character somewhat superior to, that is to say somewhat more specialised than that which can be obtained from a general practitioner, no means more ridiculous could be devised for the purpose than those which were adopted by the Consultative Institute. It is at last dawning upon them that to call a man a specialist or a consultant by no means makes him into either one or the other, and that to say, as is practically said by the Consultative Institute, that such a make-believe consultant is good enough for the working man, is not to treat that free and independent person with the respect which he is wont to consider his due. It appears, however, that the managers of the Hospital Saturday Fund still have faith in organisation over-riding common sense. Perhaps they know their constituents, and have faith in their general willingness to be led by the nose if only it is tickled in proper fashion. Anyway the executive committee of this fund is reported to have passed a resolution at its last meeting urging that the committee of the Consultative Institute should take immediate steps to secure a suitable successor to Dr. Irvine. Evidently the farce has been too amusing not to deserve an encore. But it is a sorry performance, and shows up the "working man" and his relations to his self-elected leaders in a most unenviable light.

